# Antimicrobial resistance patterns and biofilm formation of coagulase-negative *Staphylococcus* species isolated from subclinical mastitis cow milk samples submitted to the Onderstepoort Milk Laboratory

**DOI:** 10.1186/s12917-019-2175-3

**Published:** 2019-11-26

**Authors:** Lufuno Phophi, Inge-Marie Petzer, Daniel Nenene Qekwana

**Affiliations:** 1 Section Veterinary Public Health, Department of Paraclinical Sciences, Faculty of Veterinary Science, University of Pretoria, Private Bag X04, Onderstepoort, 0110 South Africa; 2 Department of Production Animal Studies, Faculty of Veterinary Science, University of Pretoria, Private Bag X04, Onderstepoort, 0110 South Africa

## Abstract

**Background:**

Increased prevalence of antimicrobial resistance, treatment failure, and financial losses have been reported in dairy cows with coagulase-negative *Staphylococcus* (CoNS) clinical mastitis, however, studies on CoNS infections are limited in South Africa. Therefore, the objectives of this study were to investigate the antimicrobial resistance patterns and biofilm formation in CoNS isolated from cow milk samples submitted to the Onderstepoort Milk Laboratory.

**Results:**

A total of 142 confirmed CoNS isolates were used for this study. Biofilm formation was identified in 18% of CoNS tested. *Staphylococcus chromogenes* (11%) had the highest proportion of biofilm formation followed by *S. haemolyticus* (4.0%), *S. epidermidis*, *S. hominis, S. xylosus, and S. simulans* with 1% respectively. Ninety percent (90%) of CoNS were resistant to at least one antimicrobial (AMR) and 51% were multidrug-resistant (MDR). Resistance among CoNS was the highest to ampicillin (90%) and penicillin (89%), few isolates resistant to cefoxitin and vancomycin, 9% respectively. Similarly, MDR-*S. haemolyticus* (44%), MDR-*S. epidermidis* (65%), and MDR-*S*. *chromogenes* (52%) were mainly resistant to penicillins. The most common resistance patterns observed were resistance to penicillin-ampicillin (16%) and penicillin-ampicillin-erythromycin (10%). Only 42% of biofilm positive CoNS were MDR.

**Conclusion:**

The majority of CoNS in this study were resistance to penicillins. In addition, most isolates were β-lactam resistant and MDR. Biofilm formation among the CoNS in this study was uncommon and there was no significant difference in the proportion of MDR-CoNS based on the ability to form a biofilm.

## Background

Coagulase negative *Staphylococcus* (CoNS) are among the most frequently isolated bacteria from dairy cows with clinical and subclinical mastitis [1–3]. They are emerging as opportunistic pathogens in clinical mastitis in South Africa [4] and globally [5–10]. The most commonly isolated CoNS in subclinical and clinical mastitis include *S. chromogenes, S. epidermidis, S. simulans, S. haemolyticus,* and *S. xylosus* [2, 11, 12]. Although intramammary infections caused by CoNS are usually self-limiting, however, there are reports of clinical mastitis cases that often require antimicrobial treatment [13, 14]. Penicillin antimicrobials have been reported to be effective against CoNS infections [9, 15, 16]. However, studies are reporting increasing prevalence of antimicrobial resistance in CoNS from clinical mastitis cases [17–19] including resistance to penicillin, tetracycline, lincomycin, and streptomycin [13, 20].

The increasing prevalence of resistance among CoNS could be due to the injudicious use of antimicrobials [21], the presence of penicillin binding protein 2a (PBP2a) [16, 22, 23], and *mec*A mediated oxacillin resistance [24–27]. In addition, the high prevalence of antimicrobial resistance among CoNS could be due to their ability to form a biofilm which facilitates persistent infections [9, 28, 29] and decreases susceptibility to commonly used antimicrobials [30]. The ability of *Staphylococcus* species to form biofilm has been linked to the presence of biofilm-forming genes such as *ica*A and *bap* gene [20, 30, 31], which have been isolated in CoNS species such as *S. epidermidis, S. haemolyticus, S. xylosus*, *S. chromogenes* and *S. simulans* [31]. To our knowledge, there are no studies that have reported an association between biofilm formation and high prevalence of MDR in CoNS isolated from subclinical mastitis cases in dairy cattle. In addition, no studies have been published on the antimicrobial resistance patterns of CoNS from dairy cattle in South Africa.

Thus, the aim of this study was to investigate the antimicrobial resistance patterns and biofilm formation of CoNS isolated from cow milk samples submitted to the Onderstepoort milk laboratory, Department of Production Animal Studies, South Africa. We hypothesize that CoNS with biofilm formation isolated from subclinical mastitis cases have an increased prevalence of resistance to commonly used antimicrobials. In addition, it also possible that these isolates are β-lactam and multidrug-resistant (MDR).

## Results

### Coagulase negative *Staphylococcus* species

A total of 142 CoNS isolates were tested for biofilm formation and antimicrobial resistance, the majority of the isolates tested were *S. chromogenes* (70%; 100/142), followed by *S. epidermidis* (12%; 17/142), *S. haemolyticus* (11%; 16/142), *S. simulans* (2%; 3/142),*S. xylosus* (2%; 3/142), *S. hominis* (1%; 1/142), *S. hyicus* (1%; 1/142) and *S. scuiri* (1%; 1/142).

Of the isolates tested, 18% (26/142) formed biofilm. Among biofilm producing isolates, 11% were *S. chromogenes*, followed by *S. haemolyticus* (4%) and *S. epidermidis* (1%). No biofilm formation was identified in *S. scuiri* and *S. hyicus* (Table 1).

**Table 1:** Biofilm formation of coagulase-negative *Staphylococcus* species (*n* = 142) isolated from cow milk samples at the Onderstepoort milk laboratory, 2017

Organism	Tested	Number of Biofilm forming Isolates			Total Biofilm formation			
		Weak	Moderate	Strong	Number	Percent	95%	CI^a^
*S. chromogenes*	100	6	7	3	16	11	7	18
*S. epidermidis*	17	1	0	0	1	1	0	4
*S. haemolyticus*	16	4	2	0	6	4	18	61
*S. hominis*	1	0	0	1	1	1	1	7
*S. scuiri*	1	0	0	0	–	–	–	–
*S. xylosus*	3	1	0	0	1	1	0	4
*S. simulans*	3	1	0	0	1	1	0	4
*S. hyicus*	1	0	0	0	–	–	–	–

^a^95% CI = 95% confidence interval

In total, 90% (128/142) of CoNS were resistant to at least one antimicrobial (AMR), with most isolates resistant to ampicillin (63%) and penicillin (63%). Few CoNS isolates were resistant to cloxacillin (16%), cefoxitin (9%), and vancomycin (9%). More than half (51%, 73/142) of CoNS were multidrug resistant (MDR). Multidrug resistant CoNS were mainly resistant to penicillin (88%), ampicillin (85%) and erythromycin (64%) (Table 2). The most common resistant patterns identified among CoNS were penicillin-ampicillin (16%; 17/106) and penicillin-ampicillin-erythromycin (10%; 11/106).

**Table 2:** Antimicrobial resistance patterns of coagulase-negative *Staphylococcus* species isolated from cow milk samples at the Onderstepoort milk laboratory, 2017

Group	Antimicrobial	AMR-CoNS^b^ (*n* = 142)			MDR-CoNS^c^ (*n* = 73)		
		Percent	95%	CI^a^	Percent	95%	CI^a^
Lincosamide	Clindamycin	11	7	17	19	12	30
Penicillins	Penicillin	63	55	70	88	78	93
	Ampicillin	63	55	71	85	75	91
	Cloxacillin	16	11	23	30	21	41
Tetracyclines	Oxytetracycline	11	7	17	21	13	31
Phenicols	Chloramphenicol	6	3	11	10	5	18
Flouroquinolone	Ciprofloxacin	8	4	13	12	7	22
Cephalosporin	Cefoxitin	9	5	15	18	11	28
Glycopeptide	Vancomycin	9	5	15	16	10	27
Aminoglycoside	Streptomycin	30	23	38	47	36	58
Macrolide	Erythromycin	49	41	58	64	53	74

^a^95% CI = 95% confidence interval

^b^AMR-CoNS = Antimicrobial resistance of coagulase negative *Staphylococcus* species resistant to at least one antimicrobial

^c^MDR-CoNS = Multidrug resistance coagulase negative *Staphylococcus species*

Among biofilm positive CoNS, 92% (24/26) were resistant to at least one antimicrobial, half of the isolates were resistant to erythromycin (54%) and penicillin (50%). While 42% (11/26) of biofilm positive isolates were MDR. Biofilm positive isolates with MDR were resistant to penicillin (82%), erythromycin (73%), ampicillin (64%) and streptomycin (55%) (Table 3).

**Table 3:** Antimicrobial resistance patterns of biofilm positive coagulase-negative *Staphylococcus* species isolated from cow milk samples at the Onderstepoort milk laboratory, 2017

Group	Antimicrobial	AMR-CoNS^b^ biofilm positive (*n* = 24)			MDR-CoNS^c^ biofilm positive (*n* = 11)		
		Percent	95%	CI^a^	Percent	95%	CI^a^
Lincosamide	Clindamycin	21	9	41	36	15	65
Penicillins	Penicillin	54	35	72	82	52	95
	Ampicillin	42	25	61	64	35	85
	Cloxacillin	4	1	20	9	2	38
Tetracyclines	Oxytetracycline	8	2	26	18	5	48
Phenicols	Chloramphenicol	8	2	26	18	5	48
Flouroquinolone	Ciprofloxacin	13	4	31	18	5	48
Cephalosporin	Cefoxitin	8	2	26	18	5	48
Glycopeptide	Vancomycin	8	2	26	9	2	38
Aminoglycoside	Streptomycin	46	28	65	55	28	79
Macrolide	Erythromycin	58	39	76	73	43	90

^a^95% CI = 95% confidence interval

^b^AMR-CoNS = Antimicrobial resistance of coagulase negative *Staphylococcus* species resistant to at least one antimicrobial

^c^MDR-CoNS = Multidrug resistance of coagulase negative *Staphylococcus species*

### Staphylococcus chromogenes species

Ninety-three percent (93/100) of *S. chromogenes* were resistant to at least one antimicrobial. Isolates were mainly resistant to ampicillin (66%), penicillin (63%) and erythromycin (54%). Low resistance was observed to vancomycin (11%) and cefoxitin (6%). Multidrug resistant *S. chromogenes* (52%; 52/100) exhibited a high prevalence of resistant to penicillin (87%), ampicillin (87%), erythromycin (69%) and streptomycin (54%) (Table 4). The most common resistant patterns among *S. chromogenes* were the penicillin-ampicillin-erythromycin (91%) and penicillin-ampicillin pattern (71%). Among biofilm positive *S. chromogenes,* 50% (8/16) were MDR.

**Table 4:** Antimicrobial resistance pattens of *Staphylococcus chromogenes* isolated from cow milk samples at the Onderstepoort milk laboratory, 2017

Group	Antimicrobial	AMR-*S. chromogenes*^*b*^ (*n* = 100)			MDR-*S. chromogenes*^*c*^ (*n* = 52)		
		Percentage	95%	CI^a^	Percentage	95%	CI^a^
Lincosamide	Clindamycin	14	9	22	25	15	38
Penicillins	Penicillin	63	53	72	87	75	93
	Ampicillin	66	56	75	87	75	93
	Cloxacillin	14	9	22	25	15	38
Tetracyclines	Oxytetracycline	6	3	12	12	5	23
Phenicols	Chloramphenicol	4	2	10	8	3	18
flouroquinolone	Ciprofloxacin	4	2	10	6	2	16
Cephalosporin	Cefoxitin	6	3	12	12	5	23
Glycopeptide	Vancomycin	11	6	19	19	11	32
Aminoglycoside	Streptomycin	34	25	44	54	41	67
Macrolide	Erythromycin	54	44	63	69	56	80

^a^95% CI = 95% confidence interval

^b^AMR- *S. chromogenes* = *S. chromogenes* resistant to at least one antimicrobial

^c^MDR- *S. chromogenes* = Multidrug resistance *S. chromogenes*

### *Staphylococcus epidermidis* species

Overall, 94% (16/17) of *S. epidermidis* were resistant to at least one antimicrobial while 65% (11/17) were MDR. The resistance was high to penicillin (82%) and ampicillin (77%). Few isolates were resistant to vancomycin (12%), cloxacillin (35%) and cefoxitin (29%). Multidrug resistant *S. epidermidis* showed an increased prevalence of resistance to penicillin (91%) and ampicillin (91%) (Table 5). Twenty-five percent (1/4) of the biofilm positive *S. epidermidis* were resistant to at least one antimicrobial while none of the biofilm positive *S. epidermidis* isolates were MDR.

**Table 5:** Antimicrobial resistance patterns of *Staphylococcus epidermidis* isolated from cow milk samples at the Onderstepoort milk laboratory, 2017

Group	Antimicrobial	AMR*-S. epidermidis*^b^ (*n* = 16)			MDR*-S. epidermidis*^c^ (*n* = 11)		
		Percent	95%	CI^a^	Percent	95%	CI^a^
Lincosamide	Clindamycin	0	0	18	0	0	26
Penicillins	Penicillin	82	59	94	91	62	98
	Ampicillin	77	53	90	91	62	98
	Cloxacillin	35	17	59	55	28	79
Tetracyclines	Oxytetracycline	41	22	64	64	35	85
Phenicols	Chloramphenicol	17	6	41	18	5	48
flouroquinolone	Ciprofloxacin	17	6	41	27	10	57
Cephalosporin	Cefoxitin	29	13	53	45	21	72
Glycopeptide	Vancomycin	12	3	34	18	5	48
Aminoglycoside	Streptomycin	12	3	34	18	5	48
Macrolide	Erythromycin	41	22	64	45	21	72

^a^95% CI = 95% confidence interval

^b^AMR- *S. epidermidis* = *S. epidermidis* to at least one antimicrobial

^c^MDR- *S. epidermidis* = Multidrug resistance *S. epidermidis*

### Staphylococcus haemolyticus species

Eighty-one percent (13/16) of *S. haemolyticus* were resistant to at least one antimicrobial, while 44% (7/16) were MDR. The highest prevalence of resistance observed was to penicillin (56%) and few isolates were resistant to cloxacillin (13%) and cefoxitin (13%). Multidrug resistant *S. haemolyticus* were mainly resistant to penicillin (100%), ampicillin (71%), and erythromycin (57%) (Table 6). Among *S. haemolyticus* biofilm positive isolates, 100% (6/6) were resistant to at least one antimicrobial while 50% (3/6) of the isolates were MDR.

**Table 6:** Antimicrobial resistance patterns of *Staphylococcus haemolyticus*, isolated from cow milk samples at the Onderstepoort milk laboratory, 2017

Group	Antimicrobial	AMR-*S. haemolyticus*^b^ (*n* = 13)			MDR-*S. haemolyticus*^*c*^ (*n* = 7)		
		Percent	95%	CI^a^	Percent	95%	CI^a^
Lincosamide	Clindamycin	6	1	28	14	3	51
Penicillins	Penicillin	56	33	77	100	65	100
	Ampicillin	44	23	67	71	36	92
	Cloxacillin	13	3	36	29	8	64
Tetracyclines	Oxytetracycline	13	3	36	29	8	64
Phenicols	Chloramphenicol	6	1	28	14	3	51
flouroquinolone	Ciprofloxacin	19	7	43	29	8	64
Cephalosporin	Cefoxitin	13	3	36	29	8	64
Glycopeptide	Vancomycin	0	0	19	0	0	35
Aminoglycoside	Streptomycin	31	14	56	43	16	75
Macrolide	Erythromycin	25	10	50	57	25	84

^a^95% CI = 95% confidence interval

^b^AMR- *S. haemolyticus* = *S. haemolyticus* resistant to at least one antimicrobial

^c^MDR- *S. haemolyticus* = Multidrug resistance of *S. haemolyticus*

## Discussion

Coagulase negative *Staphylococcus* species (CoNS) have been reported as a cause of mastitis in dairy cattle [13] with prognosis in affected patients dependent on antimicrobial resistance profile of the isolate, the presence of virulence factors, and biofilm formation [29]. In this study, we investigated antimicrobial resistance patterns and biofilm formation of CoNS isolated from cow milk samples submitted to the Onderstepoort milk laboratory.

### Biofilm formation of CoNS

We observed a low (18%) proportion of biofilm-forming CoNS compared to other studies [20, 31, 32] For example, Tremblay et al. [31] in Canada reported 96.7% proportion of biofilm formation in CoNS from dairy cattle with mastitis. Similarly, 85.1% of CoNS from subclinical and clinical mastitis cases of dairy cattle in Argentina formed biofilm [20]. Simojoki et al. [32] in Finland also reported a high (31.3%) proportion of biofilm-forming CoNS from clinical mastitis cases in dairy cattle. The low proportion of biofilm formation of CoNS in this study compared to the above-mentioned studies may be attributed to the difference the growth media used [33], since, Tremblay et al. [31] and Simojoki et al. [32] used brain heart infusion for biofilm assay while in this study TSB was used. Nonetheless, the low proportion of biofilm formation in this study suggests that biofilm formation is not common in CoNS subclinical mastitis. Therefore, the role played by biofilm formation in the prognosis of CoNS subclinical mastitis in this study is limited. However, more studies need to be done to further explore the molecular epidemiology of biofilm formation CoNS from subclinical and clinical mastitis cases in dairy cattle, South Africa.

### Antimicrobial resistance of CoNS

A high (90%) proportion of CoNS in this study were resistant to at least one antimicrobial and no significant difference was observed in the proportion of resistance among CoNS species. Antimicrobial resistance in this study was higher than 21.4% reported in clinical mastitis cases of lactating cows in Sweden [34]. The reason for the high proportion of resistant CoNS isolates in this study is not clear. However, this could be due to selection pressure associated with injudicious use of antimicrobials for the treatment of clinical mastitis in dairy cattle from South Africa [21]. Moreover, antimicrobial drugs are easily available for farmers as over the counter medication [35]. Studies investigating antimicrobial use among farmers in the dairy industry in South Africa will be beneficial in understanding their role in antimicrobial stewardship. In addition, findings of this study suggest that interventions in the use of antimicrobial treatment among farmers in South Africa including restriction of antimicrobial use, limitation of over the counter antimicrobial, veterinary consultation, and the improvement of knowledge on antimicrobial resistance must be considered.

### Penicillin resistance

A higher (63%) proportion of penicillins resistant CoNS was observed in this study compared to that reported in clinical mastitis of dairy cattle in Finland (32%) [32], Estonia (38.5%) [36], and Zimbabwe (8%) [5]. In contrast to other studies [37, 38], we observed no difference in the proportion of penicillin resistance among CoNS species. The high proportion of penicillin resistance in this study could be due to the low affinity associated with penicillin-binding protein 2a (PBP2a) produced by *Staphylococcus* species [16, 22, 23]. In addition, this could also be due to overuse of these antimicrobials as they are readily available as over the counter antimicrobials for treatment of mastitis in dairy cattle in South Africa [39] mainly due to their narrow-spectrum activity [26, 34, 40].

### Erythromycin resistance

The resistance to erythromycin among CoNS was higher (49%) in this study than reported in subclinical mastitis of dairy cattle in Argentina (29%) [17] and in Germany (22%) [41]. In contrast, a higher (73.2%) proportion of erythromycin resistant CoNS has been reported in a study done on subclinical mastitis cases of dairy cattle in Turkey [37]. The presence of erythromycin resistance in this study may be attributed to the presence of a ribosomal methylase based resistance, encoded by *msr*A and *erm*C [42]. Furthermore, Luthje et al. [41] suggest that alteration of the ribosomal methylase activity in *Staphylococcus* spp. could lead to horizontal gene transfer. Although erythromycin is one of the antimicrobial drugs used for control of CoNS isolates in bovine mastitis, it is not used for the treatment of CoNS in South Africa. Therefore, the high prevalence of resistance observed in this study needs further investigation.

### Vancomycin resistance

Vancomycin resistance among CoNS in this study was uncommon (9%) compared to the 58.2% reported in subclinical bovine mastitis cases in Turkey [37]. In contrast, Bengtsson et al. [34] in Sweden reported no vancomycin resistance among CoNS isolated from mastitis cases in dairy cattle. Although vancomycin is not currently used for the treatment of clinical mastitis in South Africa, other peptides antimicrobial drugs such as bacitracin are used in combination intramammary applications. The presence of vancomycin resistance CoNS is of public health significance as vancomycin is the drug of choice for treatment of MRSA in human medicine [2]. Therefore, measures must be implemented including restriction on the use of peptides antimicrobial drugs in the treatment of mastitis in the dairy industry to curb the potential development of vancomycin-resistant CoNS.

### Cefoxitin and β-lactam resistance

The cefoxitin test is the preferred method for testing the CoNS for *mec*A mediated oxacillin resistance [43–45]. We observed a lower (9%) proportion of cefoxitin resistant CoNS compared to the 29.41% reported in clinical mastitis cases from dairy cattle in Tunisia [46] and 40% reported in subclinical mastitis from dairy cattle in Switzerland [47]. In addition, there was no significant difference in the proportion of cefoxitin resistant isolates within CoNS species. The presence of *mec*A mediated oxacillin resistance is suggestive of methicillin-resistant coagulase negative *Staphylococcus* species [1] and also encodes for penicillin-binding protein PBP2a. Together with the *bla*Z gene have been reported in β-lactam resistance among *Staphylococcus* species [9, 48]. Antimicrobial resistance of β-lactams is attributed to the hydrolysis and alteration of the β-lactam ring in bacteria [9] and is a common resistance mechanism to penicillins [31]. In this study, we observed a higher proportion of β-lactam resistant CoNS compared to the 23% reported in subclinical mastitis cases of dairy cows in Finland [13]. In contrast, all (100%) CoNS isolates from clinical mastitis cases of dairy cattle in Argentina were β-lactam resistant [38]. The high presence of β-lactam resistant isolates and the potential presence of methicillin resistance among CoNS are likely to result in the poor clinical outcome as these isolates are likely to be resistant to other antimicrobial groups including tetracyclines, lincosamides, aminoglycosides, and macrolides [23, 43, 49, 50].

### Multidrug resistance and biofilm formation

We observed a high (51%) proportion of MDR-CoNS compared to 45% reported in clinical mastitis cases in India [27]. The high proportion of MDR-CoNS in this study could be attributed to the presence of *mec*A mediated oxacillin resistance and a high proportion of β-lactams resistant CoNS isolates [24–27]. Nonetheless, the high occurrence of MDR-CoNS further emphasizes the need for judicious use of antimicrobial drugs in the dairy industry in South Africa.

There was no significant difference in the presence of MDR among CoNS with biofilm formation compared to those without. To our knowledge, this is the first study to compare antimicrobial resistance patterns and biofilm formation in subclinical mastitis in dairy cattle. In contrast, a study done in clinical patients in human medicine reported a high prevalence of multidrug resistance in biofilm positive CoNS compared to biofilm negative CoNS [51]. In addition, multidrug resistance among *S. aureus* from human clinical isolates in Korea was more common in isolates with biofilm formation compared to those without [52]. In human medicine, Oliveira et al. [53] reported that bacteria grown as biofilm are up to 1000 times more resistant compared to planktonic. The results of this study suggest that there is no association between biofilm formation and multidrug resistance.

The study is not without limitation, the type of growth media used in the study to assay biofilm formation may have played a role in the low proportion of biofilm identified in this study as the chemical composition of growth media have been shown to influence the expression of biofilm-forming genes in bacteria [33]. In addition, vancomycin resistance was assessed using the disk diffusion method, however, the broth dilution antimicrobial test is the preferred method for analysis of vancomycin resistance [45]. The population of isolates used in this study came from samples submitted to one laboratory. Therefore, the results of this study should not be generalized to the entire dairy industry in South Africa.

## Conclusion

Biofilm formation among the CoNS isolates in this study was uncommon and there was no significant different proportion of MDR-CoNS based on the ability to form a biofilm. The majority of CoNS in this study were resistance to penicillins. In addition, most isolates were β-lactams resistant and MDR.

The presence of high antimicrobial resistance in this study is a clinical concern and urgent actions should be taken to address the situation. Farmers in South Africa need to be made aware of the high MDR among CoNS and the need for judicious use of antimicrobials in the treatment of CoNS subclinical mastitis. The role of antimicrobial use practises in the development of resistance in subclinical mastitis in the dairy industry should be investigated. The relationship between antimicrobial resistance and biofilm formation in CoNS biofilm formation in the dairy industry is not clear and this concept needs further investigation.

## Methods

### Data source

Coagulase negative *Staphylococci* isolated from composite milk samples of subclinical mastitis cases that were submitted to the Onderstepoort milk laboratory, Faculty of Veterinary Science, University of Pretoria in 2017 were used. The laboratory receives milk samples from dairy farms across South Africa for routine diagnosis of mastitis. In total, 142 pure CoNS isolates were included in this study.

### Phenotypic identification

Milk was plated out on bovine blood tryptose agar plates (Oxoid, Quantum Biotechnologies (Pty) Ltd., South Africa). Inoculated agar plates were incubated aerobically at 37 °C (±1 °C) for 24–48 h. Presumptive *Staphylococcus* spp. colonies were initially identified based on phenotypic morphology, and biochemical tests [54]. The *Staphylococcus* isolates were confirmed using Staph API (Biomerieux South Africa (Pty) Ltd., South Africa).

### Species identification using MALDI-TOFMS

All *Staphylococcus* isolates were subjected to matrix-assisted laser desorption ionisation time-of-flight mass spectrometry (MALDI-TOF-MS) as previously described [55, 56]. Single pure colonies were transferred onto MALDI plates (Sigma Aldrich, St. Louis, MO) in duplicate and covered with 1 ll of cyano-4-hydroxycinnamic acid in an organic solution (50% acetonitrile and 2.5% tri-fluoro-acetic acid). The preparation was crystallized by air drying at room temperature. Flex Control software (Bruker Daltonics) recorded spectra set for bacterial identification. MALDI Biolayer 3.0 software (Bruker Daltonics) with an integrated pattern-matching algorithm was used to compare generated peak lists against the reference library and a score was generated based on similarity.

### Biofilm formation

The biofilm formation of CoNS isolates was investigated using the tissue culture plate method [57]. Isolates were cultured in BTA (blood tryptose agar) for 24 h at 37 °C. A loopful of a colony was then inoculated into 5 mL of trypticase soy broth (TSB) for 24 h at 37 °C. The inoculated broth was diluted using 1:100 to make a final volume of 2 ml (1.98 ml TSB: 0.02 ml inoculum). Individual wells of sterile 96 well flat bottom polystyrene tissue culture-treated plates (Sigma-Aldrich, Costar, USA) were filled with 200 μL of the diluted broth, positive control and negative control in triplicate. The plates were incubated for 24 h at 37 °C. After incubation, the plates were read to obtain optical density (OD) before washing at a wavelength of 570 nm using a micro ELISA (enzyme-linked immunosorbent assay) auto-reader (model 680, Biorad, UK). The contents of each well were then removed by gently tapping. The wells were washed with 200–300 ul of phosphate buffer saline (pH 7.4) three times while gently flicking the plates after each wash and left to dry for about 15 min.

Biofilm formed and adhered to the wells were fixed using 150 ul of (96%) methanol for 20 min, where after the contents were removed, and the plates were left to dry for 60 min. Each well was stained with 150 ul (0.2%) of crystal violet for 15 min, 150 ul of (96%) ethanol were then added into each well and covered for 30 min to elute the stain. The plates were read after washing at a wavelength of 570 nm using a micro ELISA auto-reader (model 680, Biorad, UK). This method was repeated 3 times and the OD (optical density) was averaged and subtracted from the cut off value to obtain the final OD for each isolate. A reference strain *S. epidermidis* ATCC 35984 was used as a control (Thermo Fischer).

The interpretation of the results was divided into the following categories; OD ≤ ODc (Optical density cut-off value) = no biofilm producer; ODc < OD ≤2XODc = weak biofilm producer; 2XODc < OD ≤4XODc = moderate biofilm producer; 4XODc < OD = strong biofilm producer [57]. For the purposes of analysis, weak, moderate, and strong biofilm were classified as biofilm positive.

### Antibiotic susceptibility testing

Coagulase negative *Staphylococcus* isolates were subjected to antimicrobial susceptibility testing against a panel of 11 drugs using the disc diffusion method (Kirby-Bauer method) [45] on Mueller-Hinton agar according to Clinical and Laboratory Standards Institute guidelines. The antimicrobials investigated included 10 mcg ampicillin (AMP), 10 iu penicillin G (P), 30 μg oxytetracycline (OT), 15 μg erythromycin (E), 30 μg chloramphenicol (C), 10 μg streptomycin (S), 5 μg ciprofloxacin (CIP), 30 μg cefoxitin (FOX), 10 μg vancomycin (VAN), 10 mcg clindamycin (DA) and 5 mcg cloxacillin (OB) [45]. Based on the diameter of the zone of inhibition, isolates were classified as sensitive, intermediate or resistant [45]. For the purpose of analysis, the intermediate susceptibility was considered as resistant. Isolates that were resistant to at least one antimicrobial drug were defined as “resistant” while those resistant to three or more antimicrobial categories were defined as “multidrug resistant” [58]. β-lactams resistance was classified as resistant to at least penicillins, cephalosporins or carbapenems [9, 59]. The interpretation of vancomycin was based on the criteria by Rezaeifar et al. [60].

### Data analysis

The proportions and frequencies of all the variables together with their 95% of confidence intervals were calculated and presented in table format.

## Data Availability

All the data used to support the findings of this study is available in the manuscript. Raw datasets may also be requested from the corresponding author provided that all ethical requirements have been met.

## Abbreviations

95% CI: 95% confidence interval
AMP: Ampicillin
AMR: Resistance to one antimicrobial
BTA: Blood tryptose agar
C: Chloramphenicol
CIP: Ciprofloxacin
CoNS: Coagulase-negative *Staphylococcus* species
DA: Clindamycin
E: Erythromycin
EPS: Extracellular polysaccharide
FOX: Cefoxitin
IMI: Intramammary infection
MDR: Multidrug resistance
MHA: Mueller Hinton agar
MRSA: Methicillin resistant *Staphylococcus aureus*
OB: Cloxacillin
OD: Optical density
ODc: Optical cut-off density
OT: Oxytetracycline
PEN: Penicillins
QS: Quorum sensing
S: Streptomycin
TSB: Trypticase soy broth
